# Effect of Micronized Purified Flavonoid Fraction Containing Hesperidin and Diosmin on Vincristine-Induced Neuropathy in Rats; the Role of Nitric Oxide Pathway

**DOI:** 10.5812/ijpr-154455

**Published:** 2024-11-02

**Authors:** Nikoo Abharian, Nima Naderi, Noushin Nikray, Mona Khoramjouy, Shokoofe Noori, Hamed Shafaroodi, Mehrdad Faizi

**Affiliations:** 1 Department of Pharmacology and Toxicology, School of Pharmacy, Shahid Beheshti University of Medical Sciences, Tehran, Iran; 2 Phytochemistry Research Center, Shahid Beheshti University of Medical Sciences, Tehran, Iran; 3 Department of Biochemistry, Faculty of Medicine, Shahid Beheshti University of Medical Sciences, Tehran, Iran; 4 Department of Pharmacology, School of Medicine, Tehran University of Medical Sciences, Tehran, Iran

**Keywords:** Peripheral Neuropathy, Chemotherapy, Hesperidin, Diosmin, Vincristine, Neuroprotective, Anti-inflammatory

## Abstract

**Background:**

Chemotherapy-induced peripheral neuropathy (CIPN) is a potential complication that can develop following cancer chemotherapy.

**Objectives:**

Due to limited medical interventions for the prevention and management of CIPN caused by vincristine, a painful and common complication, further research to find the mechanisms of CIPN and the development of effective preventive and therapeutic strategies is needed.

**Methods:**

We induced CIPN in male Wistar rats by administering intraperitoneal vincristine (VCR) at a dose of 0.1 mg/kg for 10 days. Treatment involved micronized purified flavonoid fraction (MPFF) containing hesperidin and diosmin (MPFF; Daflon^®^) at doses of 50, 100, and 200 mg/kg. Our investigation focused on levels of inflammatory factors (TNF-α, IL-6) in the dorsal root ganglion (DRG) tissue and included a series of behavioral tests: Von Frey, grip strength, rotarod, open field, and hot plate tests. To examine the role of the nitric oxide (NO) pathway in the effects of MPFF on VCR-induced peripheral neuropathy (VIPN), we also tested the effect of L-arginine (100 mg/kg i.p.) as a NO precursor and L-NAME (20 mg/kg i.p.) as a nitric oxide synthase (NOS) inhibitor.

**Results:**

Based on our behavioral tests, MPFF (50, 100, 200 mg/kg) effectively reduced some symptoms of VIPN, including mechanical allodynia and hyperalgesia. Notably, TNF-α and IL-6 levels in the DRG tissue of the groups treated with 100 and 200 mg/kg of MPFF showed a significant reduction in inflammatory factors. These results underscore the potential of MPFF and the role of the NO signaling pathway in alleviating neuropathic pain caused by VCR.

**Conclusions:**

Micronized purified flavonoid fraction may be an effective treatment for VIPN by reducing mechanical allodynia, thermal hyperalgesia, and TNF-α and IL-6 levels in DRG tissue. The NO pathway may play a role in the effectiveness of MPFF in treating VIPN.

## 1. Background

Chemotherapy-induced peripheral neuropathy (CIPN) is a severe and common complication that can occur following the use of various chemotherapy drugs, including platinum derivatives, taxanes, vinca alkaloids, and thalidomide. The complications of CIPN are dose-dependent and may necessitate chemotherapy dose reduction or even treatment discontinuation, ultimately impacting cancer therapy (1). The prevalence of CIPN following chemotherapy is estimated at 68.1%, and CIPN can persist for at least six months after completing chemotherapy in one-third of patients. Due to the high global prevalence of cancer, CIPN affects several million patients worldwide each year (2). Sensory symptoms of CIPN are more common and include pain, numbness, and tingling, while motor symptoms may include weakness, balance disorders, and locomotor issues. Although the exact pathology of CIPN is unclear, chemotherapy agents can impact the peripheral nervous system (PNS) and dorsal root ganglion (DRG) through mechanisms such as mitochondrial damage, oxidative stress, neuroinflammation, and changes in ion channel expression, neurotransmitters, and their receptors. These effects ultimately lead to nerve damage and neuron demyelination (3). 

The American Society of Clinical Oncology (ASCO) guidelines recommend duloxetine (DLX), a serotonin and norepinephrine reuptake inhibitor (SNRI), for managing CIPN. American Society of Clinical Oncology provides a moderate recommendation for DLX in managing CIPN, though its efficacy remains limited. Duloxetine cannot eliminate all neuropathy symptoms, and no studies have confirmed its role in preventing neuropathy complications (4).

Vinca alkaloids [vincristine (VCR), vinblastine, vinorelbine] are chemotherapeutic agents that target microtubules, preventing mitotic division and continuous cancer cell growth (5). Vincristine-induced peripheral neuropathy (VIPN) may occur in 78 - 100% of patients receiving VCR, presenting as a common and painful complication that can lead to disability, reduce quality of life, and even necessitate cessation of therapy (6). Vincristine-induced peripheral neuropathy may result from calcium homeostasis disturbances, increased expression of inflammatory factors (such as TNF-α and IL-1β), and oxidative stress (7). Vincristine-induced peripheral neuropathy affects sensory, motor, and autonomic nerves, leading to symptoms such as paresthesia, numbness, pain, difficulty walking, decreased reflexes, balance disorders, muscle cramps, visual impairment (including blurred vision or transient blindness), constipation, urinary incontinence or retention, and orthostatic hypotension. Due to the beneficial effects of flavonoids on the circulatory system and their anti-inflammatory and antioxidant properties (8), the micronized purified flavonoid fraction (MPFF) Daflon^®^, containing 90% diosmin and 10% hesperidin, has been used to treat conditions such as chronic venous disease (9). Diosmin is obtained from citrus pericarp or through the oxidation of hesperidin. It is distinguished from hesperidin by a double bond between two carbon atoms in the C ring. Due to its antioxidant, anti-cancer, anti-diabetic, and antibacterial properties, diosmin has potential benefits for treating various diseases (10, 11). Diosmin also exhibits anti-inflammatory activity and can reduce the levels of several inflammatory factors (12). Hesperidin, a well-known flavonoid, possesses potent antioxidant, anti-inflammatory, and neuroprotective properties and is extracted from citrus fruits, tea, and olive oil. Research indicates that hesperidin may be beneficial in improving nervous system disorders due to its neuroprotective effects (13, 14). In the chronic constriction injury (CCI) neuropathy model, induced by chronic compression of the sciatic nerve in rats, hesperidin attenuates both mechanical and thermal hyperalgesia. Additionally, the simultaneous use of hesperidin with diosmin reduced mechanical and thermal hyperalgesia. Treatment with hesperidin also decreased the levels of inflammatory factors (TNF-α, IL-1β, IL-6) in the sciatic nerve and spinal cord of rats in the CCI model. Besides reducing hyperalgesia, hesperidin can attenuate central sensitivity to neuropathic pain by influencing the TNF-α pathway (15).

In the CCI rat model, diosmin reduces hyperalgesia through opioid and D2 receptors and decreases the pro-inflammatory cytokines IL-1, TNF-α, and IL-6. In pain and inflammation caused by lipopolysaccharide (LPS) in mice, diosmin attenuated the production of inflammatory factors (IL-1β, TNF-α, IL-6) and oxidative stress (16). Nitric oxide (NO), a neurotransmitter in the central nervous system (CNS), is produced from the amino acid L-arginine by the enzyme nitric oxide synthase (NOS). L-NAME is among the compounds used to inhibit NOS in studying the NO-cGMP pathway. Excessive NO production caused by changes in NOS isoforms contributes to neurotoxicity and neuropathic pain. Following inflammation and the activation of inflammatory factors such as tumor necrosis factor α, glial cell death occurs via inducible NOS. Studies have highlighted the pathogenic role of NO in nerve damage, with the primary target of NO-mediated toxicity in inflammatory neuropathy being axon damage and myelin destruction in sensory neurons.

Currently, there is no suitable treatment for VIPN, and we have chosen to evaluate the efficacy of MPFF on VIPN for the first time.

## 2. Objectives

Given the high prevalence of the painful complication of CIPN in cancer patients undergoing chemotherapy, along with the limited effective treatment options and the proven antioxidant, anti-inflammatory, and neuroprotective properties of the flavonoids diosmin and hesperidin, the aim of this research is to evaluate the effect of MPFF (containing diosmin and hesperidin) on VIPN.

## 3. Methods

### 3.1. Animals

The study utilized male Wistar rats weighing 250 to 300 g (12 - 14 weeks of age) obtained from the Pasteur Institute, Tehran, Iran. The animals had unrestricted access to food and water and were maintained on a 12-hour light/dark cycle. All experiments and animal care were conducted in accordance with the ethical standards set by the Institutional Animal Care and Use Committee (IACUC) of Shahid Beheshti University of Medical Sciences, with approval code IR.SBMU.PHARMACY.REC.1402.135.

### 3.2. Drugs and Chemicals

Micronized Purified Flavonoid Fraction (Daflon^®^), containing 90% diosmin and 10% hesperidin, along with DLX powder, was suspended in distilled water, while VCR (Sobhan Oncology Co., Iran) was dissolved in sterile normal saline (0.9% NaCl). Vincristine was administered intraperitoneally (i.p.), and MPFF and DLX were administered via oral gavage for 10 days.

### 3.3. Experimental Design

This study consisted of eight distinct groups, with each group comprising eight randomly selected male Wistar rats. The groups included the control group of healthy rats, which received 0.1 mL/kg of normal saline intraperitoneally (i.p.) daily. The control group was the only group in this study that did not receive VCR. The VCR group, designated as the neuropathy group, received VCR (0.1 mg/kg) intraperitoneally for 10 days (17) without any additional treatment. All treatment groups received VCR (0.1 mg/kg) intraperitoneally for 10 days. The experimental groups consisted of the MPFF 50 group, which received VCR + 50 mg/kg MPFF; the MPFF 100 group, which received VCR + 100 mg/kg MPFF; the MPFF 200 group, which received VCR + 200 mg/kg MPFF; the MPFF 100 + L-A group, which received VCR + 100 mg/kg MPFF + 100 mg/kg L-arginine (18); the MPFF 100 + L-NAME group, which received VCR + 100 mg/kg MPFF + 20 mg/kg G-nitro-L-arginine-methyl ester (L-NAME) (19); and the DLX group, which received VCR + 30 mg/kg DLX (20). All dosages were selected based on a review of the literature and our pilot study findings.

### 3.4. Induction of Peripheral Neuropathy by VCR-Induced Peripheral Neuropathy

Peripheral neuropathy was induced in the animals through daily intraperitoneal administration of VCR (0.1 mg/kg) for 10 days (21).

### 3.5. Experimental Design

On days 0, 7, and 10 of VCR treatment, the open field, rotarod, Von Frey, and grip strength tests were conducted. Testing began with the open field test, followed by the grip strength, rotarod, and Von Frey tests, with at least a one-hour interval between each test. The hot plate test was conducted only on the 10th day. After completing the experiments on the last day of the study, the animals were anesthetized using ketamine (100 mg/kg, i.p.) and xylazine (10 mg/kg, i.p.). The bilateral lumbar DRG (L4 - L6) was then surgically dissected. To evaluate the expression of TNF-α and IL-6 using the ELISA method, the dissected tissues were frozen in liquid nitrogen for 10 minutes and subsequently stored at -80°C.

### 3.6. Behavioral Tests

#### 3.6.1. Von Frey test

Mechanical allodynia was assessed using the Von Frey test with filaments of varying thicknesses, based on the method of Sahranavard et al. (22). Briefly, animals were placed on top of a metal mesh within a plastic box, and the plantar paw of each animal was stimulated using Von Frey filaments. The response was defined as the paw withdrawal threshold. Each filament was applied five times to each paw, and if the animal responded three out of five times, it was recorded as a response.

#### 3.6.2. Grip Strength Test

In this research, we evaluated the muscle strength of the animals using the grip strength test (Borj Sanat Azma, Iran). In this experiment, while the animal grasps the metal wire of the device with its front limb, it is gently pulled back by the tail. The force exerted by the animal as it pulls on the device is recorded. For each rat, the experiment was repeated three times with a 5-minute interval between trials. Finally, the calculated average force was normalized to the animal's body weight (in mN/g) (23).

#### 3.6.3. Rotarod Test

The rotarod test assesses the ability of rats to maintain balance and coordination while walking on a rotating rod. In this experiment, the animals were placed on a rotating rod (3 cm diameter) at a fixed speed of 6 rpm, and the latency time for each animal to fall from the rod was recorded. This experiment was repeated three times for each rat with a 5-minute interval between trials, and the recorded times were averaged for each animal. The cut-off time was set at 120 seconds (24).

#### 3.6.4. Open Field Test

The general motor activity of the animals was assessed using the open-field test. Each animal was placed in a 40 × 40 × 40 cm Plexiglas box, filmed from above by a digital camera, and observed for 10 minutes. After removing each animal from the box, the testing environment was cleaned with 70% ethyl alcohol. At the end of the experiments, the videos recorded by the camera were analyzed using Ethovision XT software (Noldus, The Netherlands), and the total distance moved (in cm) was reported (25).

#### 3.6.5. Hot Plate Test

Thermal hyperalgesia was assessed using the hot plate test, which involves heating a metal plate with an electric current (Borj Sanat Azma, Iran). At a temperature of 52.5 ± 0.5°C, rats were placed on the hot plate, and the latency to respond by either paw licking, hind paw shaking, or jumping was recorded as the response and reported as the latency to withdraw. To prevent injury, a 10-second cut-off time was implemented (26).

### 3.7. Role of Nitric Oxide Pathway

To investigate the involvement of the NO pathway in the effect of MPFF on VIPN, neuropathic rats were treated with MPFF (100 mg/kg), L-arginine (100 mg/kg i.p.), a NO precursor, and G-nitro-L-arginine-methyl ester (L-NAME) (20 mg/kg i.p.), a NOS inhibitor, before conducting the grip strength and hot plate tests.

### 3.8. Enzyme-Linked Immunosorbent Assay

To assess the impact of MPFF (Daflon^®^) on VCR-induced neuroinflammation, levels of TNF-α and IL-6 were measured in DRG tissue using an enzyme-linked immunosorbent assay (ELISA) kit (Sigma, St. Louis, USA). The color intensity was read using a spectrophotometer (Perkin Elmer, USA) at 450 nm.

### 3.9. Statistical Analysis

The data obtained from the experiments were analyzed using GraphPad Prism 8, and results were presented as mean ± SEM in behavioral tests and mean ± SD in enzyme-linked immunosorbent assay. For the Von Frey, grip strength, rotarod, and open field tests, differences between group means were analyzed using a two-way analysis of variance (ANOVA) with Bonferroni post hoc tests. For the hot plate test and the comparison of inflammatory factor levels, differences between groups were analyzed using one-way ANOVA with Tukey’s post hoc multiple comparison test. A P-value of less than 0.05 was considered statistically significant.

## 4. Results

### 4.1. Behavioral Tests

**Figure 1.**
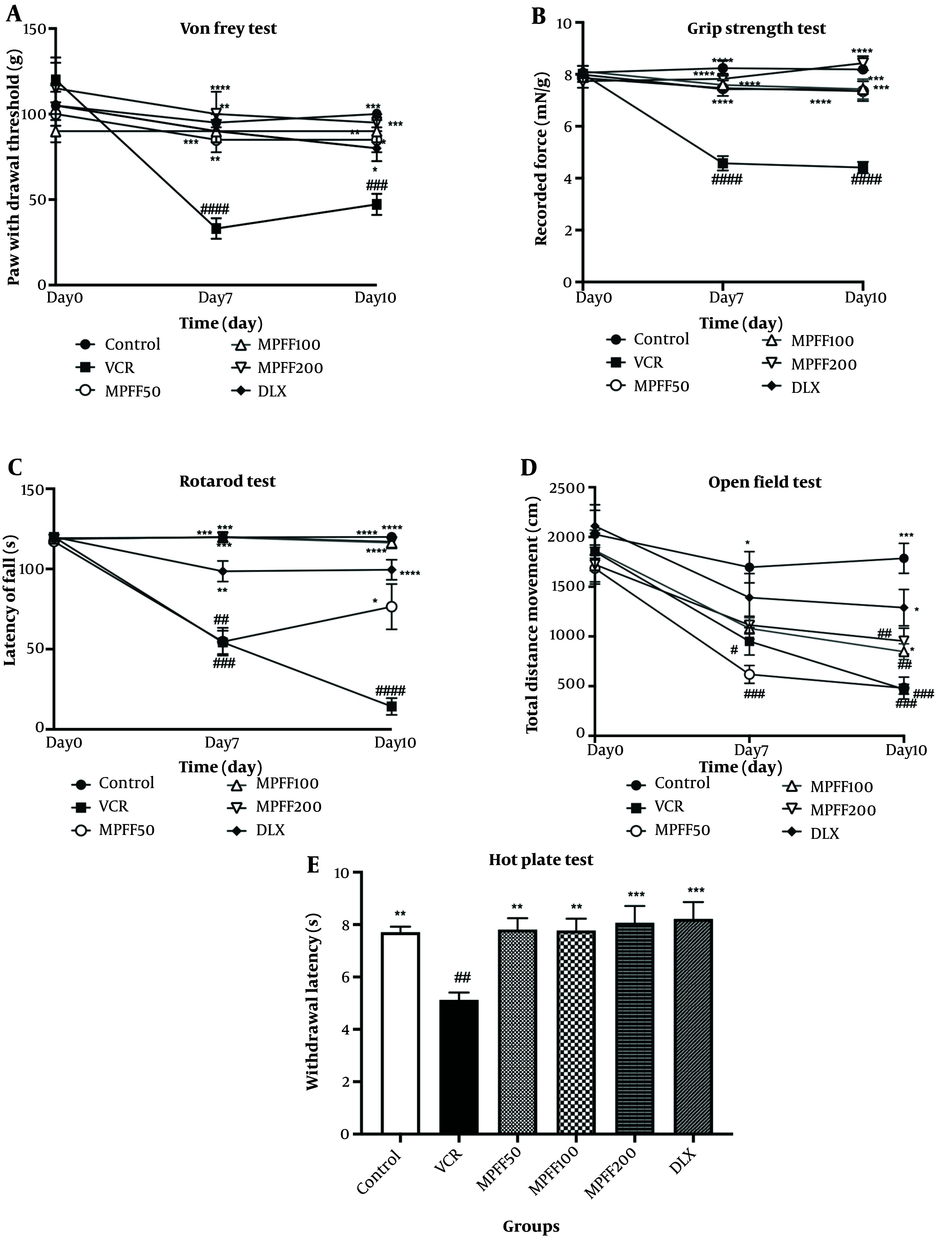
Effect of micronized purified flavonoid fraction (MPFF); A, on allodynia in Von Frey test; B, on muscle strength in grip strength test; C, on latency to fall from rotarod test; D, on total distance moved in open field test; E, on withdrawal latency hot plate test, in vincristine (VCR)-induced neuropathy model. Data are presented as mean ± SEM and n = 8 in all groups. * P < 0.05; ** P < 0.01; *** P < 0.001; and **** P < 0.0001 show significant differences compared to the VCR group. # P < 0.05; ## P < 0.01; ### P < 0.001; and #### P < 0.0001 indicates a significant difference compared to the control group. VCR = group received VCR without treatment, MPFF 50 = group received VCR + 50 mg/kg MPFF, MPFF 100 = group received VCR + 100 mg/kg MPFF, MPFF 200 = group received VCR + 200 mg/kg MPFF and duloxetine (DLX) = group received VCR + 30 mg/kg DLX.

The paw withdrawal threshold in the Von Frey test was measured on days 0, 7, and 10 of the experiment. As shown in Figure 1A, the VIPN model was established, as the group receiving VCR on days 7 and 10 showed significant differences (P < 0.0001 and P < 0.001, respectively) compared to the control group. On days 7 and 10, all treatment groups, including the three MPFF groups and DLX, displayed significant differences compared to the VCR group (P-values are shown in the Figure 1). 

The grip strength test was conducted on days 0, 7, and 10 to assess muscle strength differences between groups. As shown in Figure 1B, on days 7 and 10, the VCR group indicated a significant difference compared with the control group (P < 0.0001), confirming the establishment of the VIPN model, similar to the Von Frey test. On days 7 and 10, the mean force applied in all treatment groups, including the MPFF groups and DLX, was significantly different compared with the VCR group (P-values are shown in the Figure 1). 

The rotarod test was performed on days 0, 7, and 10 to compare motor coordination and balance among the experimental groups. The latency time to fall from the rotarod is shown in Figure 1C. The VCR group showed significant differences on days 7 and 10 (P < 0.001 and P < 0.0001, respectively) compared to the control group. The MPFF 50 group did not show a significant difference from the VCR group on day 7; however, MPFF 100, MPFF 200, and DLX groups displayed significant differences compared to the VCR group on day 7 (P < 0.01 for the DLX group and P < 0.001 for MPFF 100 and MPFF 200). On day 10, the latency to fall from the rotarod in all treatment groups was significantly higher than the VCR group (P < 0.05 for MPFF 50 and P < 0.0001 for MPFF 100, MPFF 200, and DLX).

Locomotor activity was assessed by the open field test on days 0, 7, and 10, and the total distance moved by rats was reported in Figure 1D. The total distance moved in the VCR group was significantly reduced on days 7 and 10 compared to the control group (P < 0.05 for day 7 and P < 0.001 for day 10). Compared to the VCR group, MPFF at doses of 50 and 100 mg/kg did not increase the total distance moved on either day 7 or day 10. However, on day 10, MPFF at a dose of 200 mg/kg and DLX significantly increased the total distance moved compared to the VCR group (P < 0.05 for both treatment groups).

Thermal hyperalgesia was assessed using the hot plate test on the tenth day of the experiment. Withdrawal latencies are shown in Figure 1E. The latency in the VCR group was significantly lower than in the control group (P < 0.01), indicating that the VIPN model was successfully established. Micronized purified flavonoid fraction at all three doses and DLX were able to reverse hyperalgesia compared to the VCR group (P-values are shown in the Figure 1). The withdrawal latency in these treatment groups was not significantly different from the control group.

### 4.2. Effects of L-arginine and L-NAME in Grip Strength and hot Plate Test

**Figure 2.**
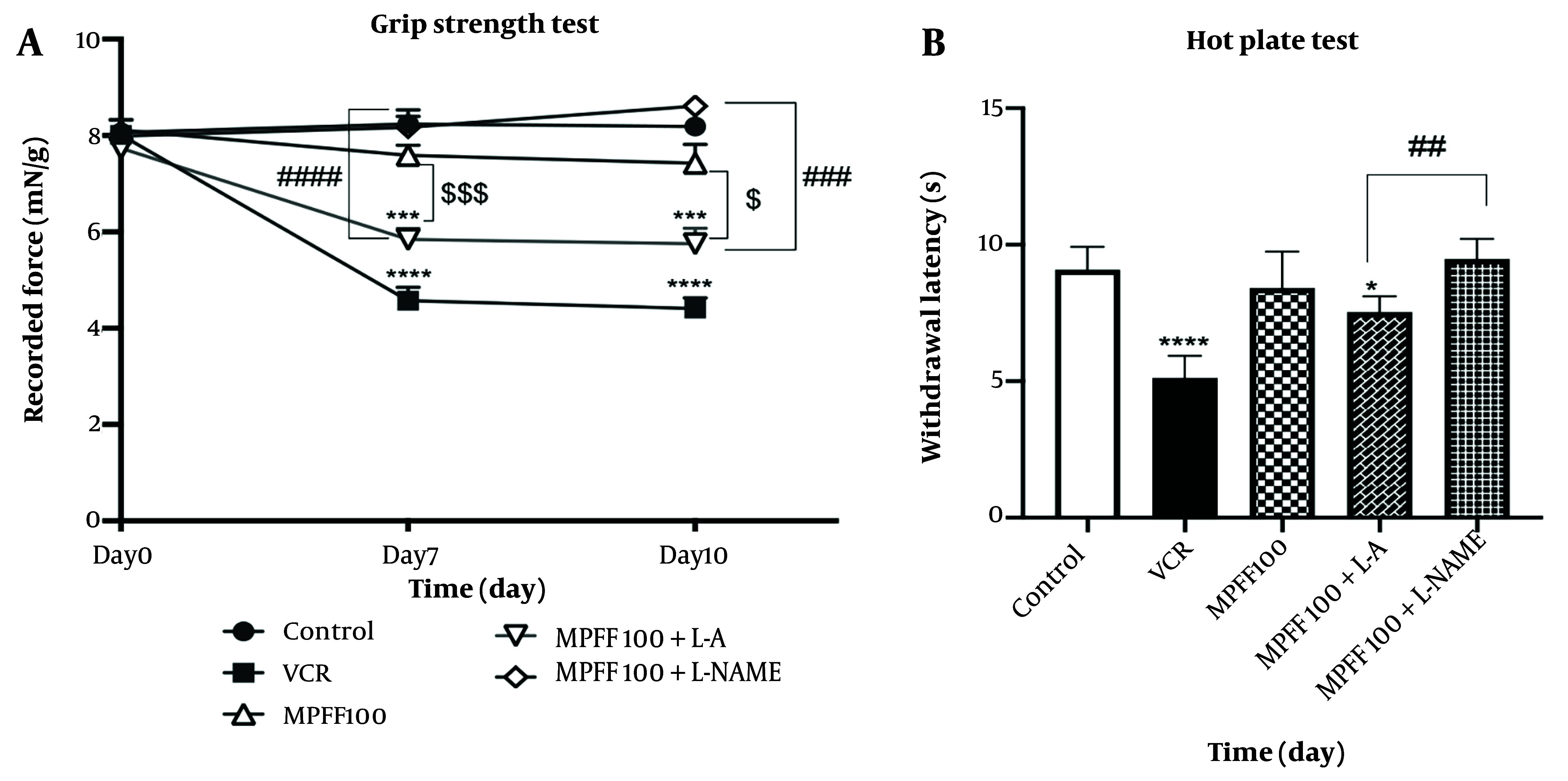
Effect of L-arginine and L-NAME on the efficacy of micronized purified flavonoid fraction (MPFF). A, effect of MPFF and MPFF plus L-arginine or L-NAME on grip strength in vincristine (VCR)-induced neuropathy model. *** P < 0.001; and **** P < 0.0001 show significant differences compared to the control group. $ P < 0.05; and $$$ P < 0.001 showed significant differences when the MPFF 100 group was compared to the MPFF 100 + L-arginine group. ### P < 0.001 and #### P < 0.0001 showed significant differences when the MPFF 100 + L-NAME group was compared to the MPFF 100 + L-arginine group; B, effect of MPFF and MPFF plus L-arginine or L-NAME on withdrawal latency in VCR-induced neuropathy model using hot plate test. * P < 0.05 and **** P < 0.0001 show significant differences compared to the control group. ## P < 0.01 indicates a significant difference when MPFF 100 + L-arginine compared to MPFF 100 + L-NAME. Data are presented as mean ± SEM and n = 8 for each group. VCR = group received VCR without treatment, MPFF 100 = group received VCR + 100 mg/kg MPFF, MPFF 100 + L-A = group received VCR + 100 mg/kg MPFF + 100 mg/kg L-arginine and MPFF 100 + L-NAME = group received VCR + 100 mg/kg MPFF + 20 mg/kg L-NAME.

L-arginine and L-NAME were used to investigate the role of the NO pathway in the effect of MPFF on VIPN. As shown in Figure 2A, on days 7 and 10, grip strength was reduced in comparison to the control group (P < 0.0001 for both days). MPFF at a dose of 100 mg/kg increased grip strength on both days 7 and 10 compared to the VCR group (P < 0.001). In the group that received both L-arginine and 100 mg/kg MPFF, grip strength was significantly lower than in the group that received 100 mg/kg MPFF alone (P < 0.001 on day 7 and P < 0.05 on day 10). Comparing grip strength in the group that received both L-arginine and 100 mg/kg MPFF with the group that received L-NAME and 100 mg/kg MPFF shows that grip strength on both days 7 and 10 was higher in the group that received L-NAME (P < 0.0001 for day 7 and P<0.001 for day 10).

L-arginine and L-NAME were also used to investigate the role of the NO pathway in the effect of MPFF on thermal hyperalgesia, assessed using the hot plate test. As shown in Figure 2B, the latency in both the VCR group and the group that received L-arginine and 100 mg/kg MPFF was significantly lower than in the control group (P < 0.0001 and P < 0.05, respectively). Comparing the withdrawal latency between the group that received both L-arginine and 100 mg/kg MPFF and the group that received L-NAME and 100 mg/kg MPFF shows that the latency was higher in the group that received L-NAME (P < 0.01).

### 4.3. Enzyme-Linked Immunosorbent Assay

**Figure 3.**
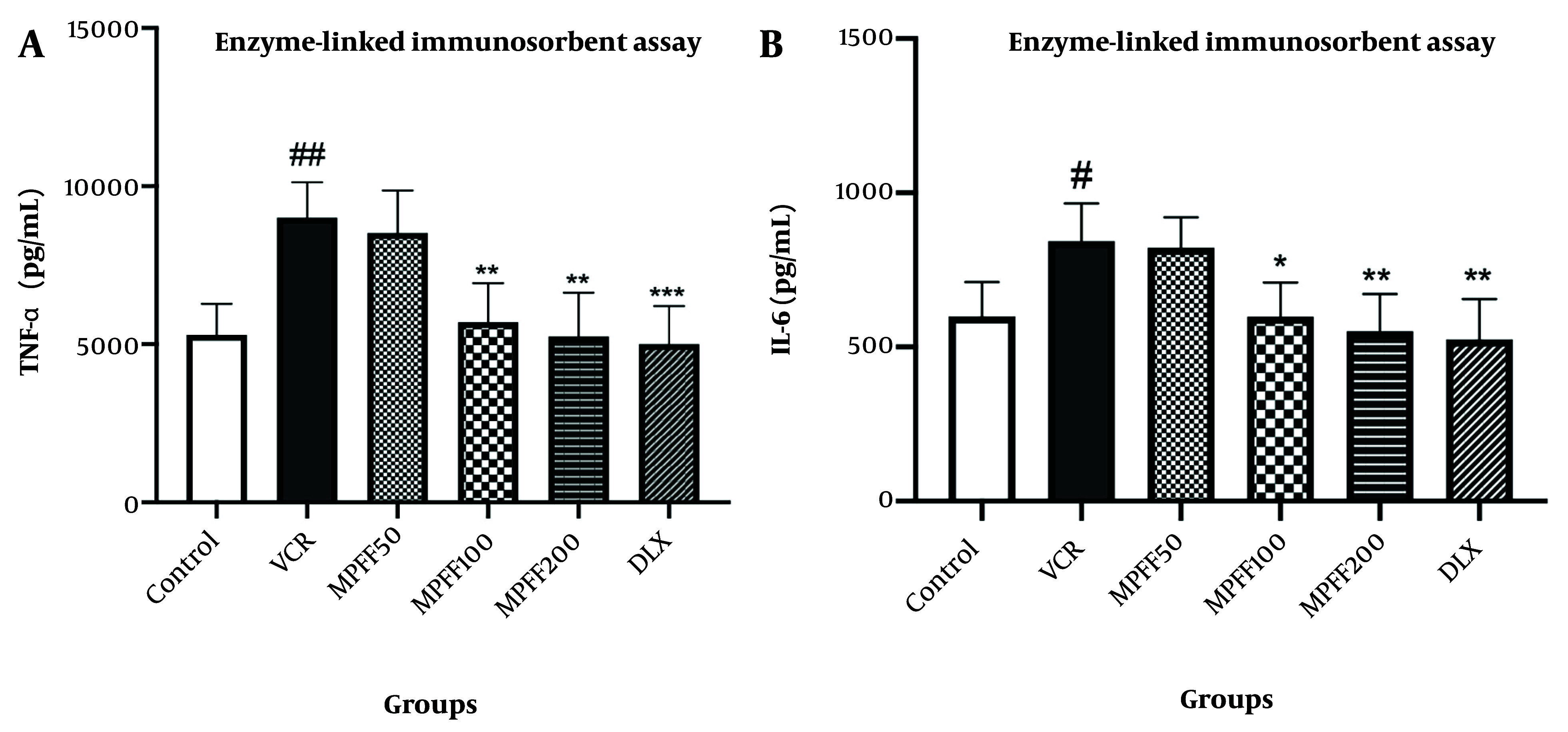
A, The effect of micronized purified flavonoid fraction (MPFF) on the level of TNF-α in vincristine (VCR)-induced neuropathy model; B, the effect of MPFF on the level of IL-6 in VCR-induced neuropathy model. Data are presented as mean ± SD and n = 5 in all groups. * P < 0.05; ** P < 0.01; and *** P < 0.001 show significant differences compared to the VCR group. # P < 0.05; ## P < 0.01 shows a significant difference compared to the control group. VCR = group received VCR without treatment, MPFF 50 = group received VCR + 50 mg/kg MPFF, MPFF 100 = group received VCR + 100 mg/kg MPFF, MPFF 200 = group received VCR + 200 mg/kg MPFF, duloxetine (DLX) = group received VCR + 30 mg/kg DLX.

The levels of TNF-α in the DRG are shown in Figure 3A. TNF-α levels in the VCR group were significantly higher than in the control group (P < 0.01). Additionally, the MPFF 100 and MPFF 200 groups showed a significant reduction in elevated TNF-α levels compared to the VCR group (P < 0.01). TNF-α levels in the group that received DLX were significantly lower than in the VCR group (P < 0.001). The effect of 100 mg/kg of MPFF in reducing TNF-α levels in the DRG was comparable to the effect of 30 mg/kg of DLX.

The levels of IL-6 in the DRG are shown in Figure 3B. IL-6 levels in the VCR group were significantly higher than in the control group (P < 0.05). Moreover, the MPFF 100 and MPFF 200 groups showed a significant reduction in elevated IL-6 levels compared to the VCR group (P < 0.05 and P < 0.01, respectively). IL-6 levels in the group that received VCR and DLX were significantly lower than in the VCR group (P < 0.01). The effect of 100 mg/kg of MPFF on reducing IL-6 levels was comparable to that of 30 mg/kg of DLX.

## 5. Discussion

Despite the life-saving benefits of an anticancer treatment regimen containing VCR, unwanted effects such as paresthesia, allodynia, and hyperalgesia which may occur in the early stages of treatment or even years after therapy ends can lead to a reduction in drug dosage or discontinuation of treatment (27). Allodynia and hyperalgesia are manifestations of VIPN. In this study, the experimental model of VIPN was well developed based on results obtained from the Von Frey test (to assess allodynia) and the hot plate test (to assess hyperalgesia). Rats treated with VCR for ten days exhibited a reduced threshold for responding to physical and thermal stimuli, along with lower locomotor activity and decreased grip strength. These findings align with our previous study (22).

Due to a limited understanding of the causes and pathogenesis of VIPN and its varied symptoms, appropriate prevention and treatment strategies are limited, despite VIPN’s high prevalence (28). Recent studies have identified inflammation in the nervous system, along with nitrosative and oxidative stress, as critical factors in the development of VIPN. Pharmacological studies in animal models have demonstrated that antioxidant and anti-inflammatory drugs can effectively treat VIPN (5, 28). Excessive secretion of inflammatory mediators in the nervous system, including the spinal cord and DRG, contributes to the induction and persistence of neuropathy. However, nerve cells can mitigate nerve damage and neuropathy by producing anti-inflammatory cytokines such as transforming growth factor-β (TGF-β), IL-4, IL-10, and IL-13. Consequently, targeting inflammatory markers presents a promising therapy with fewer undesired effects for treating peripheral neuropathy (29).

Plant secondary metabolites are beneficial in treating numerous chronic diseases with minimal side effects. Flavonoids, a class of plant secondary metabolites, possess various properties, including analgesic, anti-inflammatory, and antioxidant functions. They can suppress the production of pro-inflammatory factors dependent on NF-kB, intercellular adhesion molecules, and vascular endothelial growth factors. Additionally, flavonoids activate transcription factors associated with anti-inflammatory responses, such as nuclear factor erythroid 2-related factor 2 (Nrf2). Consequently, flavonoids may offer a safe and effective treatment option for neuropathic pain and oxidative stress (30). Hesperidin, a flavanone glycoside found in citrus fruits, exhibits anti-inflammatory, antioxidant, and neuroprotective effects. In various CNS disorder models, hesperidin has demonstrated valuable neuroprotective, antioxidant, and anti-inflammatory properties (14). In rats with CCI and streptozotocin (STZ)-induced neuropathic pain, hesperidin administration (50 or 100 mg/kg) has shown significant benefits in alleviating mechanical and thermal hyperalgesia (31). Additionally, hesperidin (50 mg/kg) increased antioxidant enzyme activity (such as superoxide dismutase, glutathione peroxidase, and catalase) and effectively reduced cisplatin-induced neurotoxicity and tissue damage (32). At doses of 100 or 200 mg/kg, hesperidin can reduce elevated levels of NF-kB, IL-1β, TNF-α, cyclooxygenase-2 (COX-2), nNOS, Janus tyrosine kinase (JAK2), and signal transducers and activators of transcription 3 (STAT3) caused by paclitaxel in rats. Additionally, it enhances Bcl-2 levels and decreases levels of caspase-3 and Bax, thereby protecting against paclitaxel-induced apoptosis in the sciatic nerve (33).

Diosmin, an unsaturated glycoside, is derived from the dehydrogenation of hesperidin and is found in various citrus plants (34). A wide range of in vitro and in vivo studies have confirmed numerous pharmacological properties of diosmin, including antioxidant, antibacterial, anti-diabetic, anti-inflammatory, anti-mutagenic, anti-cancer, anti-ulcer, neuroprotective, and hepatoprotective effects (11). Diosmin has proven effective in ameliorating lipopolysaccharide (LPS)-induced pain and inflammation in mice by reducing Nuclear factor kappa-light-chain-enhancer of activated B cell (NF-κB) activation and the production of inflammatory cytokines (IL-1β, TNF-α, and IL-6). In STZ -induced diabetic neuropathy in rats, diosmin treatment (at doses of 50 and 100 mg/kg) has shown improvements in thermal hyperalgesia, allodynia, walking performance, and levels of antioxidant defense factors (35). In animal studies on CCI-induced neuropathy, diosmin has effectively ameliorated hyperalgesia (18, 20), and it can enhance the anti-hyperalgesic effect of hesperidin in rats with CCI (36).

Considering the beneficial antioxidant, anti-inflammatory, and neuroprotective properties of diosmin and hesperidin, this research utilized doses of 50, 100, and 200 mg/kg of MPFF (Daflon^®^), which consists of the flavonoids diosmin and hesperidin, as a treatment for VIPN. The results from behavioral tests illustrate that MPFF effectively reduced symptoms of VCR-induced neuropathy (mechanical allodynia and hyperalgesia), with no significant difference between the 100 and 200 mg/kg doses. Micronized purified flavonoid fraction also improved the reduced locomotor activity and grip strength caused by VCR. These findings are consistent with previous studies on CCI-induced neuropathy.

Due to its neuroprotective effect, diosmin has demonstrated benefits in mitigating memory and cognitive impairment caused by scopolamine and traumatic brain injury in mouse models. It is also effective in alleviating hyperalgesia, allodynia, movement disorders, and neuropathic pain in diabetic animals or CCI neuropathy models (11). With its neuroprotective properties, hesperidin also positively impacts a range of nervous system disorders, including mental disorders, neuroinflammation, demyelination diseases, and neurodegenerative diseases (14).

Flavonoids can significantly inhibit inflammatory factors such as IL-6, TNF-α, and NF-kB. Their anti-inflammatory effect provides valuable support in reducing neuropathic pain (30). The anti-hyperalgesic effect of hesperidin in the CCI neuropathy model in rats is attributed to the inhibition of pro-inflammatory cytokines TNF-α and IL-6. Similarly, the anti-hyperalgesic function of diosmin is associated with a decrease in TNF-α and IL-6 levels (37). In this study, we found that the administration of MPFF reduces vincristine-induced increases in inflammatory factors (IL-6 and TNF-α) in the DRG. Therefore, we conclude that the reduction of IL-6 and TNF-α in the DRG may contribute to the beneficial effects of MPFF observed in the hot plate test.

The influence of flavonoids on NO levels may vary depending on research methodology, flavonoid structure, and the presence of inflammatory or oxidative conditions. Under oxidative stress or inflammation, flavonoids can inhibit the NF-kB pathway and reduce the expression of inducible nitric oxide synthase (iNOS), thereby preventing the spread of inflammation (38). Nitric oxide, a neurotransmitter in the CNS, is derived from the amino acid L-arginine through the NOS enzyme. L-NAME is one agent used to inhibit NOS in studies on the NO-cGMP pathway (39). In mice, hesperidin exhibits an antidepressant effect by inhibiting the L-arginine-NO-cGMP pathway, while diosmin demonstrates anti-inflammatory and antioxidant properties in human chondrocyte cells by reducing mRNA expression of iNOS, COX-2, IL-1β, MMP-3, and MMP-9 (40). In LPS-induced oxidative stress in rats, MPFF modulates antioxidant enzymes and decreases iNOS levels in brain and liver tissues. In this research, a NO precursor (L-arginine) and a NOS enzyme inhibitor (L-NAME) were used to investigate the involvement of the NO signaling pathway in the effects of MPFF. Based on the hot plate and grip strength test results, the NO signaling pathway may play a role in MPFF’s effectiveness in alleviating VCR-induced neuropathic pain (41).

This study has some limitations, including the lack of histopathological and toxicological analyses. We suggest further studies in the future to examine the effects of MPFF on DRG structure and the myelination of motor and sensory neurons.

### 5.1. Conclusions

Considering the beneficial properties of the flavonoids hesperidin and diosmin in reducing neuropathic pain and hyperalgesia, their critical role in various neurological disease models, and the positive outcomes of behavioral tests in this study, it is reasonable to propose that MPFF may be effective in relieving the symptoms and pain associated with VIPN. The potential effects of MPFF on VIPN may be linked to the NO pathway. However, further investigation is essential to fully understand the mechanisms underlying VIPN formation and the complexity of these processes, encouraging additional research and exploration in this area.

## Data Availability

The dataset presented in the study is available on request from the corresponding author during submission or after publication.
